# *NTRK* fusion in Japanese colorectal adenocarcinomas

**DOI:** 10.1038/s41598-021-85075-y

**Published:** 2021-03-11

**Authors:** Yuya Yamashiro, Taisei Kurihara, Takuo Hayashi, Yoshiyuki Suehara, Takashi Yao, Shunsuke Kato, Tsuyoshi Saito

**Affiliations:** 1 Department of Human Pathology, Juntendo University School of Medicine, 2-1-1, Hongo, Bunkyo-ku, Tokyo, Japan; 2 Department of Medicine for Orthopaedics and Motor Organ, Juntendo University Graduate School of Medicine, 2-1-1, Hongo, Bunkyo-ku, Tokyo, Japan; 3 Department of Medical Oncology, Juntendo University School of Medicine, 2-1-1, Hongo, Bunkyo-ku, Tokyo, Japan

**Keywords:** Cancer, Genetics, Gastroenterology, Medical research, Molecular medicine, Oncology, Pathogenesis

## Abstract

*NTRK* fusion-positive tumors are known to be highly sensitive to TRK inhibitors, such as larotrectinib and entrectinib. Therefore, identification of patients who can potentially benefit from these inhibitors is important; however, the frequency of *NTRK* fusions in Japanese patients with colorectal cancer (CRC) is unknown. We performed pan-TRK staining using TMA-based immunohistochemistry (IHC) on samples from 971 consecutive Japanese CRC cases from a single institution. Positive cases were further analyzed using NanoString and subsequent targeted RNA sequencing. We found three positive cases using TRK-IHC. Furthermore, the Nanostring assay supported the presence of *NTRK* fusion in these cases. Subsequent targeted RNA-sequencing and RT-PCR revealed two cases with *TPM3-NTRK1* and one with *TPR-NTRK1*. The TNM stages of these cases were stage I, stage IIA, and stage IIIB, and two showed microsatellite instability-high status. Next-generation sequencing analysis using Cancer hotspot panel revealed *TP53* and *SMAD4* mutations in separate cases. IHC of β-catenin did not show nuclear accumulation. We found three cases (0.31%) of CRC with *NTRK1* fusion among 971 consecutive Japanese CRC cases. No potential driver alterations other than *NTRK* fusion were identified in these three patients.

## Introduction

The neurotrophin kinase (*NTRK*) genes *NTRK1*, *NTRK2* and *NTRK3* encode the tropomyosin receptor tyrosine kinases TRKA, TRKB and TRKC, respectively. These receptor tyrosine kinases function during normal neuronal tissue development and maintenance. Genome-wide and RNA sequence analyses have identified gene rearrangements involving each of the *NTRK* genes in malignancies, including colorectal carcinomas (CRCs). To date, various kinds of *NTRK* fusions have been reported in CRCs, including *LMNA-NTRK1*^1–4^, *PLEKHA-NTRK1*^5^, *ETV6-NTRK3*^3^, *SCYL3-NTRK1*^6^, *TPM3-NTRK1*^4,7^, *TPR-NTRK1*, and *EML4-NTRK3*^4^. These fusion genes are thought to promote tumor growth by driving expression of constitutively active fusion proteins through the activation of downstream signal transduction pathways, such as phosphoinositide 3-kinase/protein kinase B (PI3K-AKT) and mitogen-activated protein kinase (MAPK) signaling cascades^8–10^. *NTRK* fusions have been reported to be infrequent in common cancers^11^, and pathognomonic in specific rare cancers, such as infantile fibrosarcoma^12,13^ and mammary analog secretory carcinoma^14^. *NTRK* fusion-positive tumors are reported to be highly sensitive to TRK inhibitors such as larotrectinib and entrectinib; therefore, identification of patients who can potentially benefit from these inhibitors is important. In CRC, the frequency of *NTRK* fusion is reported to be as high as 0.3%^4^, although its frequency in Japanese patients with CRC is unknown.

In this study, we performed pan-TRK staining using tissue microarray (TMA)-based immunohistochemistry (IHC) in a large cohort of consecutive Japanese CRC samples. Positive cases were further analyzed using NanoString and target RNA and DNA sequencing. This is the first report to investigate the frequency of *NTRK* fusion in a large cohort of Japanese CRC patients.

## Materials and methods

### Case selection

Sequential colorectal adenocarcinoma cases were selected from the 2008–2018 pathological records at the Pathology Department of Juntendo University Hospital, Tokyo, Japan. A total of 971 colorectal adenocarcinoma cases from 956 patients (including 13 patients with synchronous double CRCs and two patients with non-synchronous double cancer) were collected. Clinicopathological information, including age, sex, tumor location, tumor depth, stromal volume, lymphatic invasion, vascular invasion, nodal metastasis and survival data, was obtained for these patients. Tissue microarray (TMA) blocks, each consisting of 2 mm cores, were made for these cases.

### Immunohistochemistry (IHC)

Immunohistochemical staining was performed using the streptavidin–biotin method^11^, with antibodies to the following: pan-TRK (clone: EPR17341; Abcam, Cambridge, MA, USA) as previously described^3^. IHC for p53 (Clone: DO-7; DAKO, Glostrup, Denmark), Ki-67 (Clone: MIB-1; DAKO, Glostrup, Denmark), and β-catenin (Clone: Rabbit Poly; DAKO, Glostrup, Denmark) were also performed. The diffuse staining pattern was judged as positive for pan-TRK, regardless of the staining intensity on TMA-based IHC. Three positive cases for pan-TRK IHC on TMA sections were also stained on the whole FFPE sections. IHC for p53 was performed on TMA sections, while IHC for β-catenin and Ki-67 was performed and evaluated using whole sections for only 3 positive cases. The p53 IHC findings were classified into overexpression, wild-type, and complete absence^15,16^. The Ki-67 labeling index was evaluated in representative areas that showed the highest immunoreactivity by counting the number of positive cells among 1000 tumor cells.

### Nanostring assay

Nanostring (NanoString Technologies, Inc., Seattle, Washington, USA) assays were performed as previously described^17,18^, to confirm that positive staining by pan-TRK immunohistochemistry led to unbalanced expression of *NTRK* gene exons, as expected of gene fusions. The custom-designed probe set contained 90 tyrosine kinase genes. For the Nanostring, 400 ng of RNA was hybridized to the probes (a reporter probe and a capture probe) at 65 °C for 18–24 h, using a thermal cycler. Samples were then inserted into the nCounter Prep Station for the removal of excessive probes, purification, and immobilization onto the internal surface of a sample cartridge for 3 h. Finally, the sample cartridge was transferred to the nCounter Digital Analyzer, where color codes were counted and tabulated for each target molecule. The expression number of the base sequence of the probe set was analyzed using nSolver software (Version 4). Using the standard deviation, a graph plotted with 2SD and 3SD lines was prepared.

### Next generation sequencing or Targeted RNA sequencing

Total RNA was extracted from formalin fixed paraffin embedded (FFPE) tissue using a Maxwell RSC RNA FFPE Kit (Promega, Madison, WI, USA). Targeted RNA sequencing was performed at RIKEN GENESIS (Tokyo, Japan). To check sample quantity and quality, the concentration of total RNA was determined with the Qubit RNA BR Assay Kit (Thermo Fisher Scientific, Waltham, MA, USA), and quality was examined using an Agilent 4200 TapeStation (Agilent Technologies, Santa Clara, CA, USA). Library preparation was performed using Archer FusionPlex Lung Kit (ArcherDX, Boulder, CO, USA) and Archer Molecular Barcode (MBC) Adapters for next-generation sequencing on an Illumina platform, according to the manufacturer's protocol. In short, cDNA was first synthesized from total RNA using random priming, and RNA quality was then determined by real-time quantitative PCR. After second-strand cDNA synthesis, end repair and A-tailing steps were performed, and the MBC adapters were ligated. Two rounds of PCR with universal and gene-specific primers were then performed. The libraries were quantified using the KAPA Library Quantification Kit for Illumina (KAPA Biosystems, Wilmington, MA, USA). Next-generation sequencing was performed on an Illumina MiSeq platform, and configured to generate 151 bp paired-end reads. Data analysis, including adapter trimming, deduplication, alignment to the reference sequence, and fusion gene detection were performed in Archer Analysis 6.0.4.

### RT-PCR

Using extracted total RNA, cDNA was synthesized with SuperScript III First-Strand Synthesis System (Invitrogen/Thermo Fisher Scientific, Waltham, MA, USA) for RT-PCR^19^. RT-PCR was performed according to the manufacturer’s protocol as follows: initial denaturation at 94 °C for 2 min and 40 cycles at 94 °C for 30 s, 55 °C for 30 s, and 72 °C for 30 s, followed by 72 °C for 2 min. The PCR product was electrophoresed on a 2% agarose gel. PCR products with the appropriate and expected sizes were excised from the agarose. Purified PCR products were sequenced with dideoxynucleotides (BigDye Terminator v3.1; Applied Biosystems, Foster City, CA, USA) and specific primers, subsequently purified using a BigDye X Terminator Purification Kit (Applied Biosystems, Foster City, CA, USA), and analyzed with a capillary sequencing machine (3730xl Genetic Analyzer, Applied Biosystems). The primer sequences were designed based on the NGS results as follows: *TPM3* exon8, 5′-GATCGGTAGCCAAGCTGGAA-3 and *NTRK1* exon9, 5′-GCACAAGGAGCAGCGTAGAA-3′ for the detection of *TPM3-NTRK1* fusion; *TPR* exon24, 5′-TCTTCAGAGAGCAAGCACAGC-3′ and *NTRK1* exon10, 5′-GCACAAGGAGCAGCGTAGA-3′ for the detection of *TPR-NTRK1* fusion.

### DNA extraction and NGS by cancer hotspot panel

Genomic DNA was extracted from tumoral and corresponding non-tumoral tissues of *NTRK* fusion-positive cases using the Maxwell RSC DNA FFPE Kit—PKK Custom (Promega). We assessed DNA integrity and NGS using the Ion Ampliseq Cancer Hotspot Panel v2 (Thermo Fisher Scientific). The details of this analysis have been described previously^20^.

### MSI analysis

MSI analysis was performed as previously described^21^. Briefly, the five microsatellite loci (*BAT-25*, *BAT-26*, *D2S123*, *D5S346*, and *D17S250*) recommended in the 1997 NCI-sponsored MSI workshop were amplified in each PCR reaction. PCR products were analyzed by capillary electrophoresis (CE) using an ABI 310 Genetic Analyzer (Applied Biosystems). For interpretation purposes, microsatellite instability at ≥ 2 loci was defined as MSI-high, instability at a single locus was defined as MSI-low, and no instability at any of the loci tested was defined as MSS.

### Statistical analysis

Statistical analyses were performed using GraphPad Prism software version 7.0a (GraphPad, San Diego, CA, USA). Smirnov-Grubbs’ test was used to identify outlier.

### Ethics approval

This study was reviewed and approved by Juntendo University School of Medicine Institutional Review Board (#2017177). All experiments were performed in accordance with relevant guidelines and regulations of the institution and the Declaration of Helsinki. The informed consents were obtained from all subjects.

## Results

### Detection of TRK expression by pan-TRK IHC

The clinicopathological characteristics of the 971 CRC samples are summarized in Table 1^21^. Briefly, lymphatic invasion and vascular invasion were observed in 430 (44.3%) and 437 (45.0%) cases, respectively. The observed pathological stages were as follows: stage I, 122 patients; stage II 337; stage III 307; stage IV 189; and unknown, 3. The three unknowns were due to local resection without lymph node dissection.

**Table 1:** Clinicopathological characteristics of CRC samples.

	971 lesions, 956 patients
Age (years) (mean ± SD)	61.7 ± 11.6
**Gender**	
Male	541
Female	415
**Tumor location**	
Cecum	84
Ascending	173
Transverse	82
Descending	57
Sigmoid	244
Rectum and anal canal	331
Tumor size (mm) (mean ± SD)	45.9 ± 22.2
Preoperative therapy	31
**Macroscopic type**	
0/1/2/3/4/unclassified	13/70/773/85/7/23
**Invasion depth**	
T2/T3/T4a/T4b	177/612/139/43
**TNM stage**	
I/IIa/IIb/IIc/IIIa/IIIb/IIIc/IVa/IVb/IVc/unknown	122/301/23/12/34/232/41/132/21/37/3
Lymphatic invasion	430 (44.3%)
Venous invasion	437 (45.0%)
Lymph nodes metastasis	453 (46.7%)

*SD *standard deviation, *T2* tumor invades the muscularis propria, *T3* tumor invades through the muscularis propria into the pericolorectal tissues, *T4a* tumor invades through the visceral peritoneum (including gross perforation of the bowel through tumor and continuous invasion of tumor through areas of inflammation to the surface of the visceral peritoneum), *T4b* tumor directly invades or adheres to other adjacent organs or structures. Synchronous cancer was counted as one cancer at TNM stage, lymph node metastasis, and distant metastasis. The TNM stage of the three cases was unknown because there was no information on lymph node metastasis in local resection.

**Figure 1:**
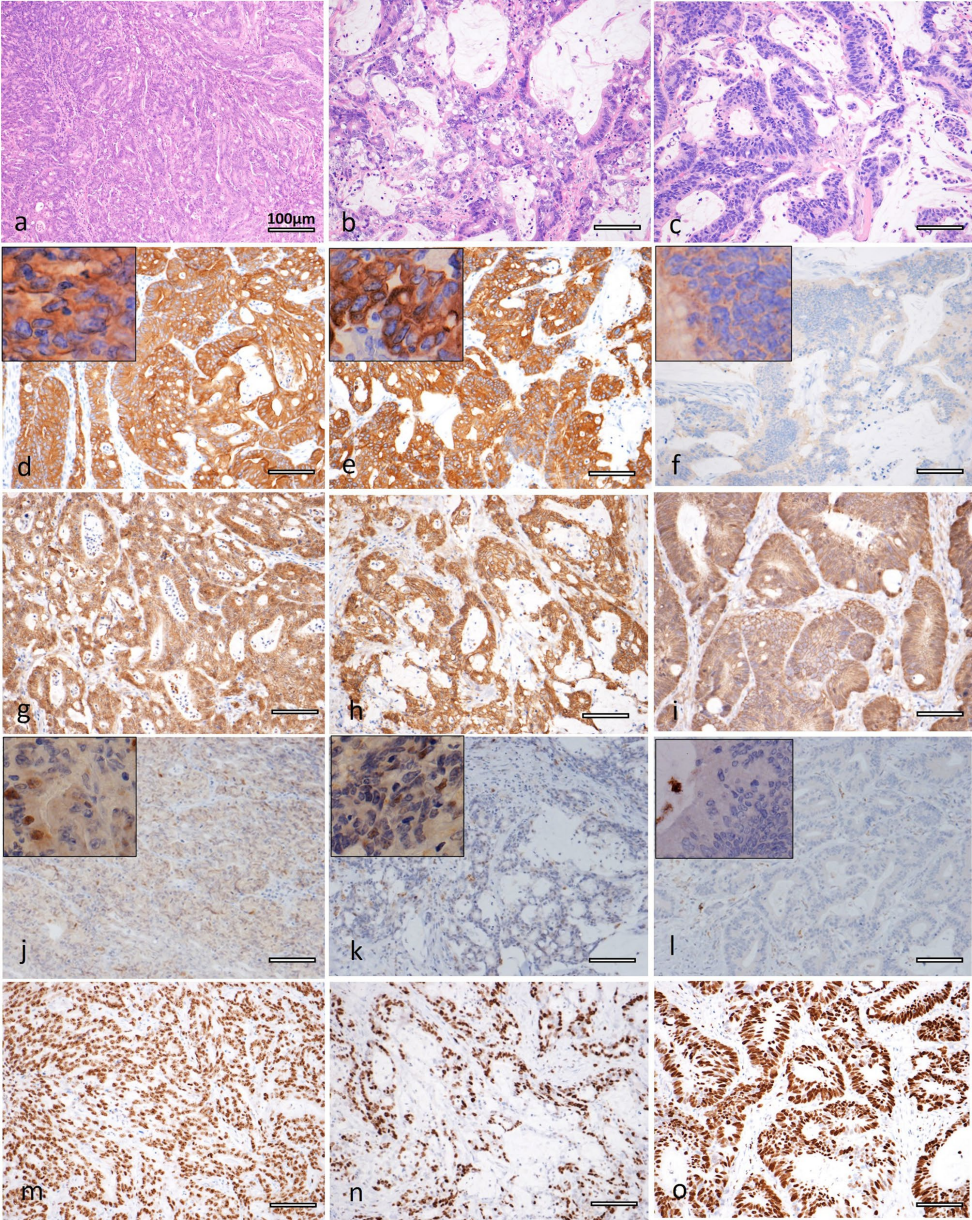
(**a**–**o**) Histological feature of Case #1 showing adenocarcinoma with a solid pattern (**a**). Histology of Case #2 showing mucinous carcinoma with a focal solid area (**b**). Histology of Case #3 showing moderately differentiated adenocarcinoma with a focal mucinous area (**c**). Pan-trk IHC images showing diffuse cytoplasmic and membranous staining for Case #1 (**d**) and Case #2 (**e**), while weak but diffuse cytoplasmic staining can be seen for Case #3 (**f**). Insets show higher magnifications. All three cases showed β-catenin membranous staining without nuclear staining (Case #1: (**g**); Case #2: (**h**); Case #3: (**i**). p53 of Case #1 (**j**) and Case #2 (**k**) showing wild type pattern, Case #3 showing completely absent pattern (**l**). Insets show higher magnifications. MIB-1 LI was high in all cases, with levels of approximately 80% in Case #1 (**m**), 50% in Case #2 (**n**) and 75% in Case #3 (**o**).

TMA-based pan-TRK IHC identified three positively stained cases. The histologies of cases #1–3 were tub2 > por1 (Fig. 1a), muc > tub2 (Fig. 1b), and tub2 > muc (Fig. 1c), respectively. Two out of three cases showed strong and diffuse membranous and cytoplasmic staining (Cases #1 and #2, Fig. 1d,e), whereas the remaining case showed weak but diffuse cytoplasmic staining (Case #3, Fig. 1f). These staining patterns were also seen in IHC analysis of whole sections.

### Nanostring assay for the pan-TRK-IHC positive samples

**Figure 2:**
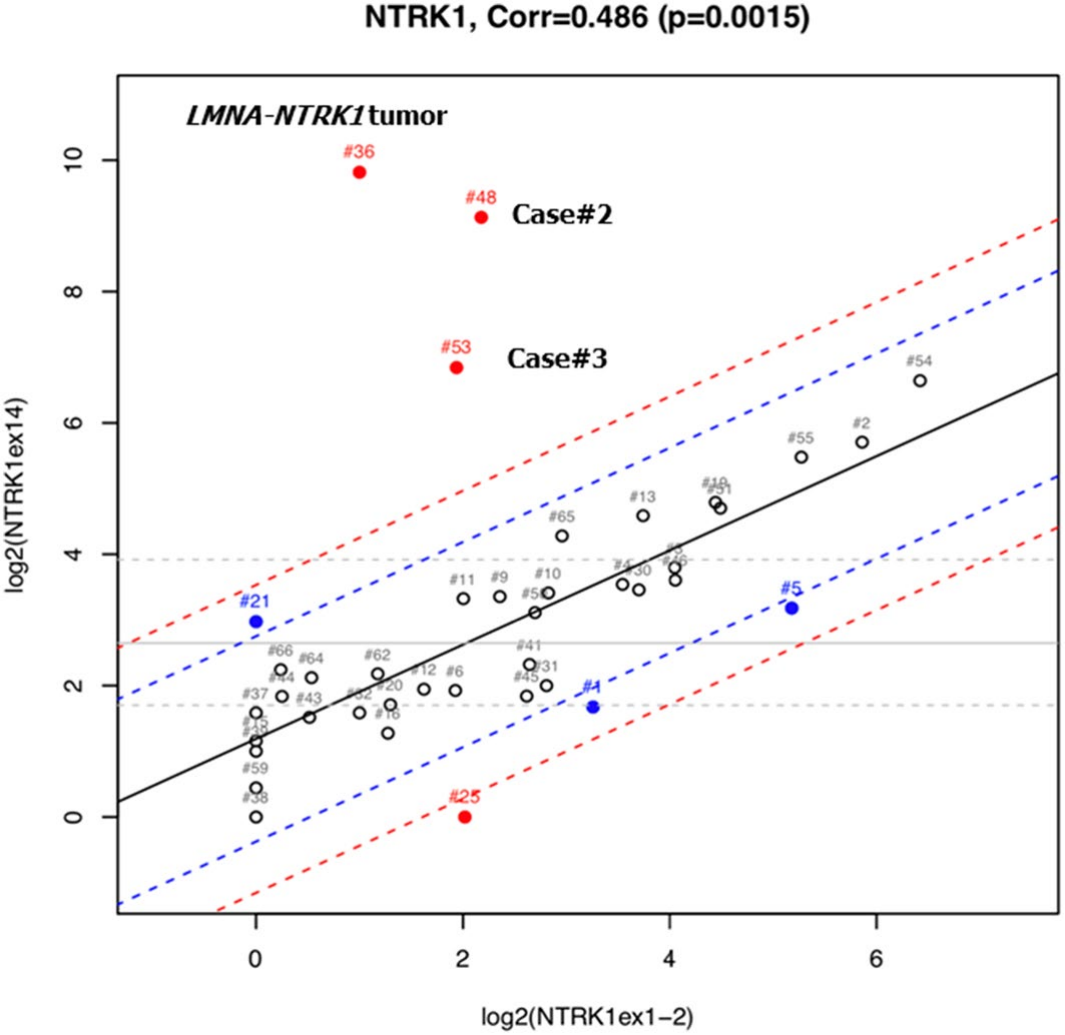
Nanostring analysis showing an imbalance of *NTRK1* gene expression between probes targeted against the 5′-side and 3′-side sequences. Smirnov–Grubbs’ test was used to identify outlier. Blue and red lines represent 2SD and 3SD, respectively. #2 and # 3 correspond to Case #2 and #3, respectively, while #36 is a positive control tumor sample previously shown to have *LMNA-NTRK1* fusion^22^. The samples other than Case #2 and #3 are not colorectal cancers. Data was analyzed with our accumulated dataset comprised of various types of malignancies. Case #21 seems to show statistically significant imbalanced expression (blotted between 2 and 3SD) of *NTRK1*. This case is an inflammatory myofibroblastic tumor previously shown to have an *ETV6-NTRK3* fusion in our previous study^20^ in which we proposed that imbalanced expression of more than 3SD would be reliable for the presence of a fusion gene.

Before sending the pan-TRK IHC-positive samples for RNA-target sequencing, a Nanostring assay consisting of 90 tyrosine kinase (TK) genes comprising *NTRK1-3* was used to evaluate the imbalance in *NTRK1-3* expression. One of the three cases (Case #1) was excluded from the statistical analysis after data normalization, probably due to poor sample quality. After the expression level of the *NTRK1* exon 14/exon1-2 was checked manually for this case, we observed more than tenfold higher expression of the *NTRK1* exon14 compared to that of *NTRK1* exon 1–2. The remaining two cases (Cases #2 and #3) showed statistically significant imbalanced expression of *NTRK1*, with expression more than triple the standard deviation (SD) of our accumulated dataset comprised of various types of malignancies, including our previously reported *LMNA-NTRK1* fusion tumor^22^ (Fig. 2). These accumulated dataset samples other than *LMNA-NTRK1* fusion tumor^22^ analyzed by Nanostring were also examined by pan-trk IHC for the detection of *NTRK* fusion tumor. Furthermore, expression level for each TK gene was also considered in addition to the imbalanced expression of TK genes to exclude the possibility of fusion formation and the subsequent overexpression.

### Targeted RNA sequencing and RT-PCR for *NTRK* fusion detection

**Figure 3:**
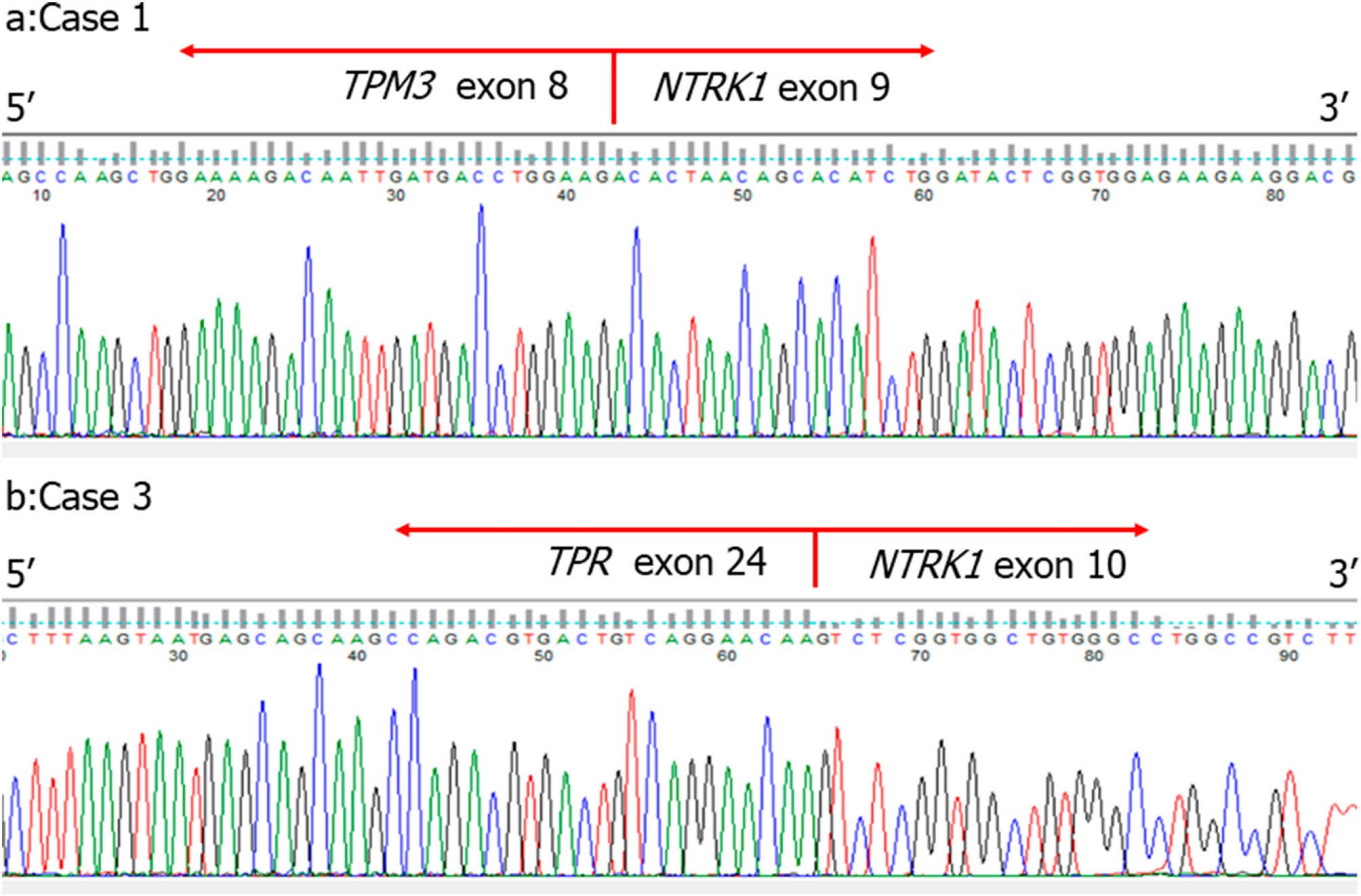
RT-PCR was performed to confirm the findings of the Archer analysis. Subsequent Sanger sequencing revealed *TPM3* (exon8)-*NTRK1* (exon9) in Cases #1 and #2, and *TPR* (exon24)-*NTRK1* (exon10) in Case #3.

RNA sequencing revealed the presence of *TPM3 (exon8)-NTRK1 (exon9)* in cases #1 and #2, and *TPR (exon24)-NTRK1 (exon10)* fusion transcript in case #3. Subsequent RT-PCR confirmed these fusion transcripts in each case (Fig. 3a,b).

### Clinicopathological characteristics of CRC with *NTRK1* fusion

Clinicopathological information on the three CRC cases harboring *NTRK1* fusions are summarized in Table 2. Briefly, the mean age of the three patients was 79 years, which was higher than that of the other CRC patients. The histologies of cases #1–3 included moderately differentiated adenocarcinoma (tub2) > poorly differentiated adenocarcinoma solid type (por1), mucinous carcinoma (muc) > tub2 and tub2 > muc, and the pathological stages were stage I, stage IIIB and stage IIA, respectively. Case #2, who presented with Stage IIIB CRC, experienced liver and lung metastasis one year after surgery and subsequently died of brain vascular disease. Case #3 received chemotherapy after surgery and exhibited no evidence of disease. For Case #1, the patient was lost to follow-up, and the prognostic information was not available at six months after surgery.

**Table 2:** Clinicopathological features of 3 CRC cases with NTRK fusions.

Case#	1	2	3
Age	84	80	73
Sex	F	M	M
Location	Ascending colon	Transverse colon	Descending colon
Histology	tub2 > por1	muc > tub2	tub2 > muc
Stage	T2N0M0 Stage I	T3N1M0 Stage IIIB	T3N0M0 Stage IIA
Prognosis	Well alive. NED (6 mos)	Liver and lung metastasis 1 year after surgery. Died of brain vascular disease	Chemotherapy after surgery. NED (100mos)
MSI	MSI-High	MSI-High	MSS
MIB-1 LI	80%	50%	75%
Pan-trk IHC staining pattern	Strong membranous and cytoplasmic staining	Strong membranous and cytoplasmic staining	Weak cytoplasmic staining
Past history	n.p	n.p	Thymoma

### Immunohistochemical characteristics of CRC with *NTRK1* fusion

All three cases with *NTRK* fusion showed β-catenin membranous staining without nuclear staining (Fig. 1g,h,i). p53 IHC showed weak to moderate nuclear staining in scattered tumor cells in Case #1 (Fig. 1j) and #2 (Fig. 1k), suggesting a wild-type staining pattern, whereas complete absence was seen in Case #3 (Fig. 1l) ^15,16^. MIB-1 LI was high in all cases, with levels of approximately 80% in Case #1 (Fig. 1m), 50% in Case #2 (Fig. 1n), and 75% in Case #3 (Fig. 1o).

### Mutation analysis by NGS

No mutations were detected in case #1. Mutations in *SMAD4* (p.Gly386Asp, c.1157G > A) and *TP53* (p.Glu294Ter, c.880G > T) were detected in cases #2 and #3, respectively. This *SMAD4* (p.G386D) mutation is described as “Pathogenic/Likely Pathogenic”.

### MSI analysis

**Figure 4:**
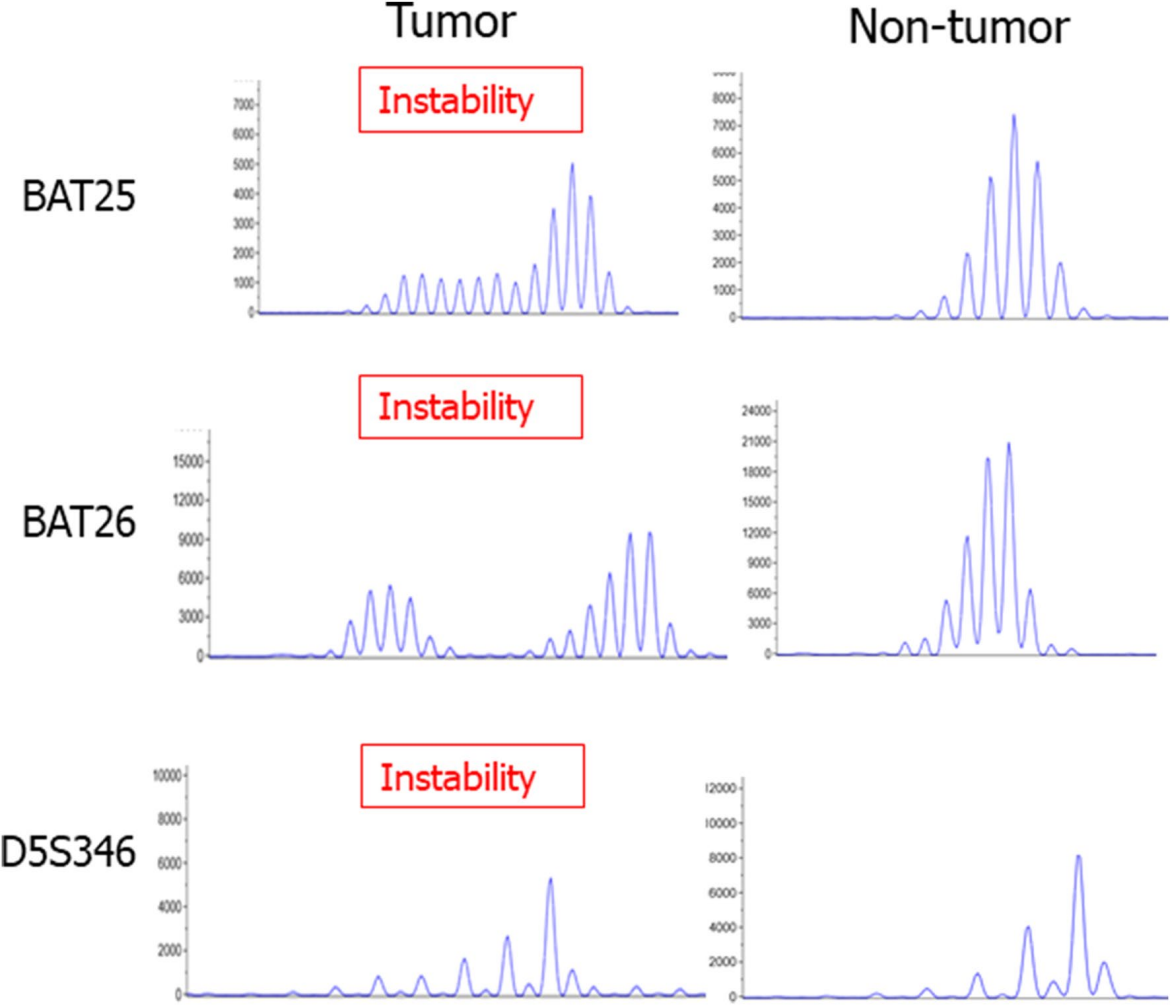
Microsatellite instability analysis. Two out of three *NTRK1* fusion CRCs showed microsatellite instability for 3 out of 5 markers. Additional peaks were observed only in the tumor-derived DNA samples for all three markers.

MSI analysis was performed for the three cases with *NTRK* fusion. Two (Cases #1 and #2) of the three cases were classified as MSI-H (Fig. 4), whereas the remaining cases were classified as MSS.

### Past and family histories

Past and family histories of neoplasms were also examined. Only Case #3 experienced thymoma, while the other two patients had no prior history of neoplasm. Regarding family history, three brothers experienced gastric cancer, leukemia, and a malignant tumor (details unknown), respectively, in Case #1. Case #2 had no particular family history. In Case #3, the patient’ s mother experienced pancreatic cancer.

## Discussion

Recent genome-wide and RNA sequence analyses have identified new fusion genes, including *NTRK* fusions, in CRC. *NTRK* fusions in CRC are extremely rare, with a reported frequency of up to 0.3%^11^; the frequency of *NTRK* fusion in Japanese patients with CRC has not been described so far. In this study, we found three CRC cases with *NTRK* fusions from examined 971 cases, based on 956 patients. Histologically, two of the three cases exhibited tubular adenocarcinoma, while the remaining case exhibited mucinous carcinoma with a tubular adenocarcinoma component. A recent study demonstrated that in CRC, *ALK*, *ROS1*, and *NTRK* rearrangements occur more frequently in elderly patients^23^, and this was also the case in our study, with the mean age of these three patients being 79 years. Furthermore, a recent study reported that most of the patients with *NTRK* fusion colon cancers were women (13/16)^4^; however, only one out of our three CRC patients with *NTRK1* fusions were women. *NTRK* fusion-positive CRCs have been reported to be located more frequently in the right side of the colon^23^, whereas another study demonstrated that CRC with *NTRK* fusion is widely distributed from the cecum to the sigmoid colon^4^. The three tumors in this study were located in the ascending, transverse, and descending colon.

We employed TMA-based pan-TRK immunohistochemistry as a prescreen for possible CRC harboring *NTRK* fusions. TMA-based immunohistochemistry is a powerful tool when dealing with thousands of clinical samples, although it has the associated risk of possibly missing positive cases due to limited information from the small cores. To evaluate the heterogeneity of pan-TRK immunohistochemistry in CRC tissue, we also performed pan-TRK IHC using whole sections in positive cases. We confirmed the same staining pattern throughout the sections. In previous reports^4^, pan-TRK IHC showed a uniform staining pattern within the same CRC sections. Based on these findings, we propose that screening for *NTRK* fusions using IHC on TMA is reliable.

Recent studies have reported that pan-TRK immunohistochemical staining in tumors with *NTRK3* fusion could be weaker than in those with either *NTRK1* or *NTRK2* fusion-positive tumors, raising the possibility of false negative findings in *NTRK3* fusion-positive tumors^24^. We also experienced a case of a soft tissue tumor with *LMNA-NTRK1* fusion showing strong cytoplasmic pan-TRK staining^22^. In this study, two *TPM3-NTRK1* tumors showed strong membranous and cytoplasmic staining, whereas the *TPR-NTRK1* tumor showed weak but diffuse cytoplasmic staining in pan-TRK IHC. These findings are consistent with those of previous reports^4,23^. The expression pattern of pan-TRK IHC may suggest a fusion partner.

The molecular pathological characteristics of the three CRC cases with *NTRK1* fusion were also assessed. The p53 IHC results for Cases #1 and #2 were wild type, while Case #3 was completely negative. Using NGS analysis, a *TP53* nonsense mutation was detected in one case (Case #3), and this case showed a complete absence pattern for p53 IHC. The remaining two cases showed a TP53 wild-type pattern by p53 IHC^15,16^, although Case #1 also showed weak cytoplasmic staining. *SMAD4* mutations were also detected in one case (case #2). Regarding this point, few *SMAD4* mutations have been reported, although mutations of other genes with a role in the transforming growth factor β (TGFβ) signaling pathway have been reported in *NTRK* fusion-positive CRCs^4^. No *APC/CTNNB1* mutations were detected, which is in accordance with the finding that none of the cases showed nuclear staining of β-catenin, a hallmark of WNT signal activation. Moreover, none of our *NTRK-*fusion-positive CRCs contained other mutations typical of CRC, such as in *KRAS* and *BRAF.* This mutually exclusive pattern between *NTRK* fusion and *KRAS/BRAF* mutation has previously been proposed^4,23^. Based on these molecular characteristics of CRC with *NTRK* fusions, the tumorigenesis of CRCs harboring activating *NTRK1* fusions could be considered different from those of conventional CRC. On the other hand, our results suggest that the TGFβ signaling pathway may be involved in a subset of these tumors. In addition, reports of MSI status in *NTRK* fusion-positive CRCs are limited^23^. A previous report on metastatic adenocarcinoma of colorectal origin demonstrated that 10 out of 13 cases with *NTRK* fusions were MSI-H^23^. A recent study demonstrated that the prevalence of *NTRK* gene fusion was around 7% in the MSI-H CRC cohort^25^. Another study also demonstrated that *NTRK* fusions were less frequent in MSS tumors than in MSI-H tumors (< 1% vs. 8%) in metastatic colon adenocarcinoma^26^. In our study, two out of three *NTRK* fusion-positive CRC patients were MSI-H, which is consistent with prior findings, although that these prior studies dealt only with metastatic CRC^23^, while our series contained consequent CRC from pStage I to pStage IV.

The prognosis of patients with CRC harboring *NTRK* fusion is not clear; however, *NTRK3* fusion-positive tumors, such as the mammary analog secretory carcinoma of the salivary gland, seem not to be aggressive^14^. Of the three CRC cases we identified with *NTRK1* fusion, one (Case #1, stage I) had been followed up for a short period, one (Case #3, stage IIA) had no evidence of recurrence or metastasis for 100 months, and the remaining patient (Case #2), with Stage IIIB CRC, died one year after surgery. This patient died of brain vascular disease, and it cannot be ruled out that he experienced malignancy-associated Trousseau syndrome. A recent study has shown that all CRC patients with *ALK-*, *ROS1-*, or *NTRK* fusions had shorter overall survival rates than fusion-negative patients^23^. Prognosis of CRC patients with *NTRK* fusion seemed to be worse or no different from that of patients with conventional CRC, although an increased sample size of CRC patients with *NTRK* fusion would be necessary to draw any significant conclusions. A recent study demonstrated that the frequency of *NTRK* fusion in CRC was 0.31%^11^. However, this number could be biased by sample selection, as patients with advanced CRC may have been selectively included in the study. Nonetheless, this frequency is similar to that of our study. As our study was a retrospective consecutive study, we could determine the frequency of CRC with *NTRK* fusion in addition to the natural history of CRC with *NTRK* fusion without specific target therapy, unlike with conventional CRC.

Finally, we found only three cases with pan-TRK staining from 971 CRC cases, including a weak staining pattern for which *NTRK* fusions were confirmed by the subsequent procedures, and the remaining cases did not show any staining. Solomon et al.^11^ showed a sensitivity of 87.5% (7/8) and a specificity of 100% (24/24) for colon cancer, and the cause of the lower sensitivity is the false negative for NTRK3 fusion tumor^11^. As a limitation of this study, our samples did not include *NTRK* fusion data; therefore, we could not evaluate the true sensitivity of this IHC-based *NTRK* fusion screening system. Although our *NTRK* fusion positive rate was similar to that of a previous study^11^, some *NTRK3* fusions tumor may have been missed due to the lower sensitivity of this test. On the other hand, another study demonstrated a CRC with *EML4-NTRK3* fusion showing weak but distinct cytoplasmic staining of pan-TRK IHC^4^. Molecular test-based screening is difficult in terms of labor and cost; thus, pan-TRK IHC could be a reliable and useful screening tool for the detection of CRC with *NTRK* fusion.
